# Tilted Material in an Optical Cavity: Light-Matter Moiré Effect and Coherent Frequency Conversion

**DOI:** 10.1021/acsphotonics.5c02118

**Published:** 2025-10-28

**Authors:** Arshath Manjalingal, Saeed Rahmanian Koshkaki, Logan Blackham, Arkajit Mandal

**Affiliations:** Department of Chemistry, 14736 Texas A&M University, College Station, Texas 77843, United States

**Keywords:** polariton, light-matter interactions, exciton-polariton, flat bands, cavity quantum electrodynamics

## Abstract

Exciton-polaritons formed inside optical cavities offer a highly tunable platform for exploring novel quantum phenomena. Here, we introduce and theoretically characterize a light-matter moiré effect (LMME) that arises when a 2D material is tilted inside a planar optical cavity, in contrast to stacking multiple layers at a twist angle as is done in forming 2D moiré heterostructures. We show that this geometric tilt produces emergent periodicity in the light-matter coupling, yielding displaced replicas of the polariton dispersion and flat bands near the Brillouin-zone center. Through time-dependent quantum dynamical simulations, we demonstrate that LMME enables coherent frequency conversion and remains robust against phonon-induced decoherence. Our findings establish LMME as a new platform for engineering polariton band structures, the generation of flat bands and performing coherent frequency conversion relevant for developing polariton-based quantum devices.

## Graphical abstract

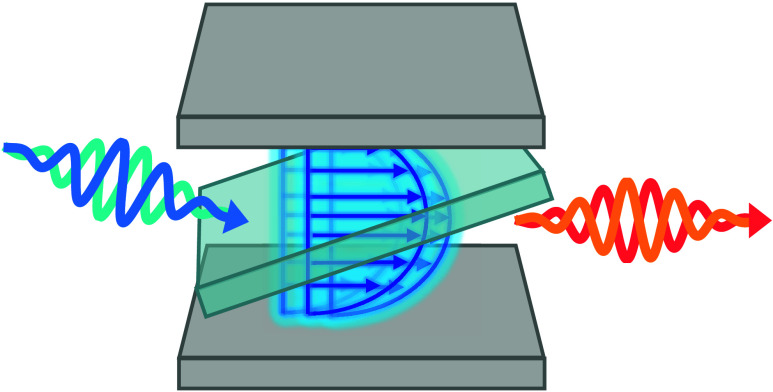

## Introduction

Strongly coupled light-matter systems support the formation of polaritons, hybrid light-matter quasiparticles, that have emerged as a versatile platform for inducing exotic physical phenomena in a highly tunable manner.^1–13^ Recent experiments demonstrate that light-matter hybrid systems can exhibit Bose–Einstein condensation at room temperature,^14–18^ polaritonic spin-Hall effect,^1,19–22^ coherent ballistic propagation at room temperature,^3,5,23–25^ and unidirectional coherence transfer,^26^ which are key ingredients for developing next-generation quantum devices. A key open question is how to harness the long-lived light-matter coherence of polaritons as a resource for quantum information science, as the interplay between spatially varying cavity fields and various material geometries remains a largely unexplored frontier for unlocking new functionalities.

In this work, we demonstrate that a moiré-like effect inside an optical cavity arises when tilting a material. Typically, the moiré effect arises when two periodic structures are overlaid at an angle, producing a new emergent periodicity,^27–29^ as illustrated in Figure 1a for twisted graphene bilayers. As schematically illustrated in Figure 1c, the relative rotation of the Brillouin zones leads to an intersection of the Dirac cones of the two graphene layers, leading to the formation of new bands.^27,29^ In this work, we find that a moiré-like effect can be achieved without multilayer stacking, but instead by tilting a single-layer material within the cavity (illustrated in Figure 1b), where the light-matter coupling generates the emergent periodic structure. We refer to this as the light-matter moiré effect (LMME). In contrast to twisted graphene, the band structure modulation in LMME is structurally different. We show that under a material tilt, replicas of the polariton band that are displaced in the reciprocal space appear (illustrated in Figure 1d) while the original polariton band remains present. Importantly, we also find that anisotropic flat bands are formed near the Brillouin-zone center. Finally, using quantum dynamics simulations, we demonstrate that LMME can be utilized to perform coherent frequency conversion and that it is robust to phonon-induced decoherence.

**Figure 1:**
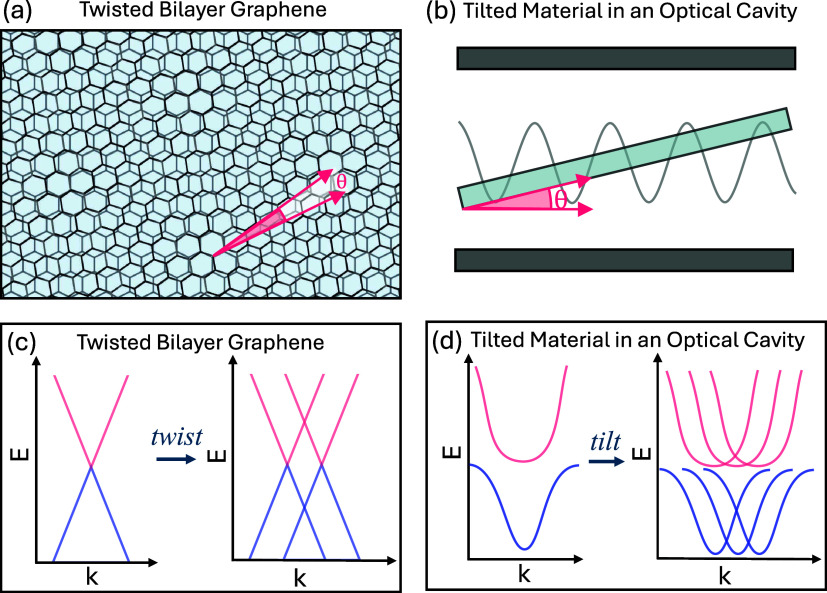
Comparison of twisted graphene and tilted material in an optical cavity (a) Illustration of a graphene bilayer offset by a twist angle. (b) Illustration of a tilted material within a Fabry-Pérot cavity coupling to cavity radiation. (c) Schematic band structure of a graphene bilayer without (left) and with (right) a twist of θ. (d) Schematic band structure of a tilted material without (left) and with (right) a tilt of θ.

It is worth noting that LMME, introduced in this work, is fundamentally different than the recently observed polaritonic spin-Hall effect, which arises due to in-plane material (refractive-index) anisotropy in filled cavities.^1,30^ While the band replicas formed due to LMME are visually similar to those formed in the polaritonic spin-Hall effect, the polaritonic spin-Hall effect do not feature the original (unshifted) polariton bands unlike in LMME. In addition to this, these displaced polariton replicas do not display circular spin polarization unlike in the spin-Hall effect. Additionally, we find that the LMME emerges only for material thicknesses much less than the cavity thickness (distance between the cavity mirrors) and that it disappears for filled cavities. Importantly, LMME allows for coherent frequency conversion, not exhibited in the polariton spin-Hall effect, with the difference in input and output photon frequencies set by the angle of tilt for the material.

## Theory

### Model

In this work, we consider a 3D setup with 2D tilted material placed inside an optical cavity as schematically illustrated in Figure 2a,e. For this system we considered a multimode Holstein-Tavis-Cummings Hamiltonian,^2,15,31–33^ which describes an exciton-polariton system beyond the long-wavelength approximation, and is expressed as

1 $${Ĥ}_{\mathrm{LM}}={Ĥ}_{\mathrm{ex}}+{Ĥ}_{\mathrm{cav}}+{Ĥ}_{\mathrm{int}}$$

where *Ĥ*_ex_ and *Ĥ*_cav_ are the bare excitonic and cavity Hamiltonians, with *Ĥ*_int_ describing the exciton-cavity interactions. In this work, we consider the 2D planar material to be multilayered, with each unit cell featuring two degenerate excited states similar to a particle in a 2D box. The bare excitonic Hamiltonian is written as

2 $${Ĥ}_{\mathrm{ex}}=\sum_{k_{\left|\right|},n_{z}}\left({\mathrm{X̂}}_{k_{\left|\right|},n_{z}}^{†}{\mathrm{X̂}}_{k_{\left|\right|},n_{z}}+{Ŷ}_{k_{\left|\right|},n_{z}}^{†}{Ŷ}_{k_{\left|\right|},n_{z}}\right)\epsilon_{k_{\left|\right|}}$$

where *X̂*_**k**_∥_, *n*_*z*__^†^ and *Ŷ*_**k**_∥_, *n*_*z*__^†^ create (Frenkel) excitons of in-plane wavevector **k**_∥_ = *k*_*x*_ *x⃗* + *k*_*y*_ *y⃗* in the *n*_*z*_th layer. Note that, $X_{k_{\left|\right|},n_{z}}^{†}=\sum_{n_{x},n_{y}}\frac{e^{-ik_{\left|\right|}\cdot R_{n}}}{\sqrt{N_{x}N_{y}}}{\mathrm{X̂}}_{n}^{†}$ and $Y_{k_{\left|\right|},n_{z}}^{†}=\sum_{n_{x},n_{y}}\frac{e^{-ik_{\left|\right|}\cdot R_{n}}}{\sqrt{N_{x}N_{y}}}{Ŷ}_{n}^{†}$ with *X̂*_**n**_^†^ and *Ŷ*_**n**_^†^ as the exciton creation operator at the lattice site **n** ≡ (*n*_*x*_, *n*_*y*_, *n*_*z*_) located at **R**_**n**_ = *n*_*x*_ *a*_*x*_ *x⃗* + *n*_*y*_ *a*_*y*_ *y⃗* + *z*_**n**_ *z⃗* with *n*_α_ ∈ {0, 1, 2, ..., *N*_α_ – 1} for α ∈{*x*, *y*, *z*}. Further, we consider a small angle of tilt θ in the *x* axis, such that $z_{n}=\left(n_{x}-\frac{N_{x}}{2}\right)b_{x}+\left(n_{z}a_{z}+\frac{L_{z}}{2}\right)$ with *b*_*x*_ = *a*_*x*_ sin­(θ). To ensure that the material is placed inside the optical cavity we impose the condition *z*_**n**_ ≡ *z*_**n**_ mod *L*_*z*_. We impose a periodic boundary condition along *x⃗* and *y⃗* direction with the in-plane box lengths *L*_*x*_ = *L*_*y*_ = *N*_*x*_ *a*_*x*_ = *N*_*y*_ *a*_*y*_. In this work, we set *a*_*x*_ = *a*_*y*_ = 12 Å, *a*_*z*_ = 30 Å, *N*_*x*_ = *N*_*y*_ = 8001 and *L*_*z*_ = 5000 Å.

**Figure 2:**
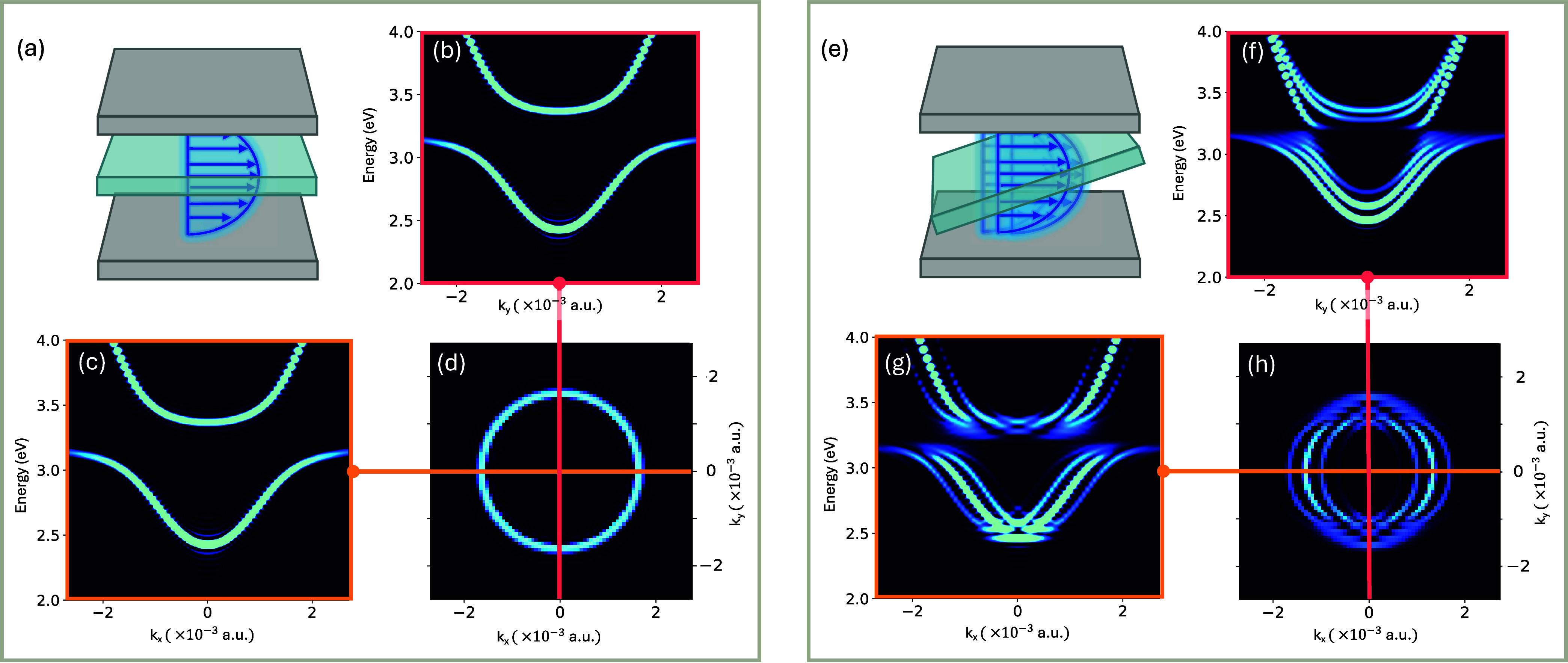
Two-dimensional band structure of a flat and tilted material (θ = 5.98°). (a) Schematic representation of a flat material inside an optical cavity, coupled to cavity radiation. (b-c) One dimensional band structures along Y and X directions, respectively for a flat material inside a cavity. (d) Two dimensional band structure at E = 3 eV of a flat material inside an optical cavity with Y (red) and X (orange) directional cuts, correlating with (b) and (c) respectively. (e) Schematic representation of a tilted material of angle θ in the X direction, inside an optical cavity, coupled to cavity radiation. (f-g) One dimensional band structures along Y and X directions, respectively for a tilted material inside a cavity. (h) Two dimensional band structure at E = 3 eV of a tilted material inside an optical cavity with Y (red) and X (orange) directional cuts, correlating with (f) and (g) respectively.

The cavity Hamiltonian *Ĥ*_cav_ is written as

3 $${Ĥ}_{\mathrm{cav}}=\sum_{k}\left({â}_{k}^{†}{â}_{k}+{\mathrm{b̂}}_{k}^{†}{\mathrm{b̂}}_{k}\right)\omega_{k}$$

where *â*_**k**_^†^ and *b̂*_**k**_^†^ creates a photon of polarization *s* (TE mode) and *p* (TM mode), respectively. Here the photonic wavevector is **k** = *k*_*x*_ *x⃗* + *k*_*y*_ *y⃗* + *k*_*z*_ *z⃗* with component along the quantization direction written as $k_{z}=\frac{m_{z}\pi }{L_{z}}$ where *m*_*z*_ ∈{1, 2, ...}. In the following work, we only consider the fifth cavity mode with *m*_*z*_ = 5, although our results are valid for arbitrary *m*_*z*_. Consequently, we denote the cavity modes only using its in-plane wavevectors, i.e., *â*_**k**_ → *â*_**k**_∥__ and *b̂*_**k**_ → *b̂*_**k**_∥__ as their *k*_*z*_ component is fixed. Further, with the periodic boundary condition along *x⃗* and *y⃗* we write $k_{x}=\frac{2\pi n_{x}}{N_{x}a_{x}}$ and $k_{y}=\frac{2\pi n_{y}}{N_{y}a_{y}}$ . The corresponding photon frequency is ω_**k**_ = *c*|**k**|/η, where η = 2.4 is the refractive index and *c* is the speed of light.

The exciton-cavity interactions Hamiltonian (*Ĥ*_int_) is written as

$${Ĥ}_{\mathrm{int}}=\sum_{n,k_{∥},j}g\sqrt{\frac{\omega_{k}}{N}}\left[{\mathrm{\mu ̂}}_{n}^{j}\cdot \left(E_{k}^{s}\left(R_{n}\right){â}_{k_{∥}}+E_{k}^{p}\left(R_{n}\right){\mathrm{b̂}}_{k_{∥}}+h.c.\right)\right]$$

Here, *N* = *N*_*x*_ *N*_*y*_ *N*_*z*_ is total number of sites and $g\propto \frac{1}{\sqrt{V}}$ (with *V* as a the quantization volume) is the light-matter coupling strength that couples the *s* and *p* photonic modes to the material dipoles **μ**_**n**_^*j*^ ∈ { **μ̂**_**n**_^*x*^, **μ̂**_**n**_^*y*^} where **μ̂**_**n**_^*x*^ = μ_*x*_ (*X̂*_**n**_ + *X̂*_**n**_^†^)*x⃗* and **μ̂**_**n**_^*y*^ = μ_*y*_ (*Ŷ*_**n**_ + *Ŷ*_**n**_^†^)*y⃗*. Here we consider an isotropic material and set μ_*x*_ = μ_*y*_ = μ and ignore the negligible dipole component in the *z⃗*- direction, as its contribution to the light-matter couplings for small angles of tilts considered in this study is vanishingly small. The spatial variation of the radiation, **E**_**k**_^*s*^(**R**_**n**_) and **E**_**k**_^*p*^(**R**_**n**_), associated with the *s* and *p* photon modes, respectively, is written as^32,34^

4 $$E_{k}^{s}\left(R_{n}\right)=\sin\left(k_{z}z_{n}\right)\left\{{\mathrm{e⃗}}_{\left|\right|}\times \mathrm{z⃗}\right\}e^{ik_{\left|\right|}\cdot R_{n}}$$

5 $$E_{k}^{p}\left(R_{n}\right)=\left[\frac{c\left|k_{\left|\right|}\right|}{\omega_{k}}\cos\left(k_{z}z_{n}\right)\mathrm{z⃗}-i\frac{ck_{z}}{\omega_{k}}\sin\left(k_{z}z_{n}\right){\mathrm{e⃗}}_{\left|\right|}\right]e^{ik_{∥}\cdot R_{n}}$$

where $e_{∥}=\frac{k_{∥}}{\left|k_{∥}\right|}$ is the unit vector along **k**_∥_. Following a set of simplifying approximations, which includes the rotating wave approximation and then using the partially Fourier transformed exciton operators ${\mathrm{X̂}}_{m,k_{y}}^{†}=\sum_{n_{y}}\frac{e^{-{\mathrm{ik}}_{y}R_{n}\cdot \mathrm{y⃗}}}{\sqrt{N_{y}}}{\mathrm{X̂}}_{n}^{†}$ and ${Ŷ}_{m,k_{y}}^{†}=\sum_{n_{y}}\frac{e^{-{\mathrm{ik}}_{y}R_{n}\cdot \mathrm{y⃗}}}{\sqrt{N_{y}}}{Ŷ}_{n}^{†}$ where **m** ≡ (*n*_*x*_, *n*_*z*_) we obtain the following expression for the light-matter interaction Hamiltonian with further details in the Supporting Information (SI),

6 $${Ĥ}_{\mathrm{int}}=\Omega_{0}\sum_{m,k_{y}}\left({\mathrm{X̂}}_{m,k_{y}}^{†}{Â}_{n_{x},k_{y}}+{Ŷ}_{m,k_{y}}^{†}{\mathrm{B̂}}_{n_{x},k_{y}}+h.c.\right)\sin\left(k_{z}z_{m}\right)$$

where $\Omega_{0}=\frac{\sqrt{\omega_{0}}\mu g}{\sqrt{N_{z}}}$ (we set $\sqrt{\omega_{0}}\mu g=0.35$ eV), $z_{m}=z_{n_{x},n_{z}}=\left(n_{x}-\frac{N_{x}}{2}\right)b_{x}+\left(n_{z}a_{z}+\frac{L_{z}}{2}\right)$ and we introduce the elliptically polarized photonic operators *Â*_**k**_∥__ and *B̂*_**k**_∥__ which are defined as

7 Ânx,ky=∑kxeikxnxaxNxÂk∥=∑kxeikxnxaxNxSx(k)(kyâk∥+ickzωkkxb̂k∥)

8 B̂nx,ky=∑kxeikxnxaxNxB̂k∥=∑kxeikxnxaxNxSy(k)(kxâk∥−ickzωkkyb̂k∥)

where $S_{x}\left(k\right)=1-\frac{c^{2}k_{x}^{2}}{\omega_{k}^{2}}$ and $S_{y}\left(k\right)=1-\frac{c^{2}k_{y}^{2}}{\omega_{k}^{2}}$ . This transformation allow us to write the full Hamiltonian into two noninteracting parts as

9 $${Ĥ}_{\mathrm{LM}}={Ĥ}_{\mathrm{AX}}\left(\{{\mathrm{X̂}}_{k_{∥},n_{z}},{Â}_{k_{∥}}\}\right)+{Ĥ}_{\mathrm{BY}}\left(\{{Ŷ}_{k_{∥},n_{z}},{\mathrm{B̂}}_{k_{∥}}\}\right)$$

where ${Ĥ}_{\mathrm{AX}}\left(\{{\mathrm{X̂}}_{k_{∥},n_{z}},{Â}_{k_{∥}}\}\right)$ and ${Ĥ}_{\mathrm{BY}}\left(\{{Ŷ}_{k_{∥},n_{z}},{\mathrm{B̂}}_{k_{∥}}\}\right)$ have identical structure. Due to this, we study the dynamics of our light-matter Hamiltonian by focusing only on ${Ĥ}_{\mathrm{AX}}\left(\{{\mathrm{X̂}}_{k_{∥},n_{z}},{Â}_{k_{∥}}\}\right)$ which is expressed as

10 $$\begin{array}{l} {Ĥ}_{\mathrm{AX}}=\sum_{k_{y}}{Ĥ}_{\mathrm{AX}}^{k_{y}}\\ =\sum_{k_{y}}\left[\sum_{k_{x}}\left({Â}_{k_{∥}}^{†}{Â}_{k_{∥}}\omega_{k}+\sum_{n_{z}}{\mathrm{X̂}}_{k_{∥},n_{z}}^{†}{\mathrm{X̂}}_{k_{∥},n_{z}}\epsilon_{k_{∥}}\right)+\Omega_{0}\sum_{m}\sin\left(k_{z}z_{m}\right)\left({\mathrm{X̂}}_{m,k_{y}}^{†}{Â}_{n_{x},k_{y}}+{\mathrm{X̂}}_{m,k_{y}}{Â}_{n_{x},k_{y}}^{†}\right)\right] \end{array}$$

This formulation enables us to perform independent 2D simulations at each *k*_*y*_, which can be combined to reconstruct the full 3D dynamics of the system. This Hamiltonian also illustrates that the periodicity of the system can be modulated by the angle of tilt θ, as it modifies the spatially oscillatory light-matter coupling term sin­(*k*_*z*_ *z*_**m**_), where *z*_**m**_ depends on θ. This is the origin of the LMME studied in this work. To gain intuition into the band structure modification of the polariton for tilted systems, we perform a Fourier transform along the *x⃗*- direction. This allows us to rewrite the Hamiltonian ${Ĥ}_{\mathrm{AX}}$ in the reciprocal space as,

$${Ĥ}_{\mathrm{AX}}=\underset{k_{∥}}{\sum }\left({Â}_{k_{∥}}^{†}{Â}_{k_{∥}}\omega_{k}+\underset{n_{z}}{\sum }{\mathrm{X̂}}_{k_{∥},n_{z}}^{†}{\mathrm{X̂}}_{k_{∥},n_{z}}\epsilon_{k_{∥}}\right)+\frac{\Omega_{0}}{2i}\underset{n_{z},k_{∥}}{\sum }{\mathrm{X̂}}_{k_{∥},n_{z}}^{†}\left({Â}_{k_{∥}+\Delta k_{x}}e^{i\phi_{z}}+{Â}_{k_{∥}-\Delta k_{x}}e^{-i\phi_{z}}\right)+h.c$$ 11

Here, **Δk**_**x**_ = Δ*k*_*x*_ *x⃗* = *k*_*z*_sin­(θ)*x⃗* and $\phi_{z}=k_{z}\left(\frac{-N_{x}b_{x}+L_{z}}{2}+n_{z}a_{z}\right)$ . The light-matter Hamiltonian presented in eq 11 illustrates that two photon modes differing by ± 2**Δk**_**x**_ in the *x* component of their wavevectors (given a tilt along *x*) *effectively* couple to each other through their interaction with the exciton.

## Results and Discussion

Figure 2 presents the angle-resolved polariton spectra of a 2D material within an optical cavity without a tilt (a-d) and with a tilt along the *x*-direction (e-h) computed from the photonic spectral function written as^25,35^

I(ω,k∥)=Re[limT→∞⁡∫0Tdteiωt⟨0̅|Âk∥|Ψ(t)⟩·cos(πt/2T)]

where |Ψ(0)⟩ = *Â*_**k**_∥__^†^ | 0̅⟩ with | 0̅⟩ as the vacuum state and |Ψ­(*t*)⟩ is the exciton-polariton wave function at time *t* obtained by solving the time-dependent Schrödinger equation (see details in the SI).

Figure 2a shows a schematic illustration of a 2D material (without tilt) placed inside a Fabry-Pérot cavity, where the cavity field is quantized along the *z⃗*-direction. The polaritonic dispersions along *k*_*y*_ (at *k*_*x*_ = 0) and along *k*_*x*_ (at *k*_*y*_ = 0) are displayed in Figure 2b–c, respectively. Both figures exhibit the two characteristic polariton bands formed by the coupling between a photonic band and an excitonic band,^2,15^ and they (Figure 2b,c) are identical due to the isotropic nature of the system. Consequently, Figure 2d displays a 2D cut (at *E* = 3 eV) of the polaritonic dispersion which appears as a perfect circle as expected. In Figure 2e–h, we examine exciton-polariton bands formed by coupling a single layer material, with a small tilt (θ = 5.7°), to an optical cavity (schematically illustrated in Figure 2e). Figure 2g shows the polariton dispersion along *k*_*x*_ at *k*_*y*_ = 0, which features multiple replicas of the original polaritonic bands displaced along *k*_*x*_, together with the original unshifted polaritonic bands $E_{\pm }\left(k_{x},k_{y}\right)\approx \frac{1}{2}\left[\omega_{k}+\epsilon_{0}\right]\pm \frac{1}{2}\sqrt{4\Omega_{0}^{2}+{\left(\omega_{k}-\epsilon_{0}\right)}^{2}}$ assuming ϵ_**k**_∥__ ≈ ϵ_0_ (since we focus on **k**_∥_ → 0). This polaritonic dispersion, which is the defining characteristic of the LMME, can be understood by inspecting eq 11. eq 11 shows that a photonic mode *Â*_**k**_∥__ *effectively* couples with the photonic modes *Â*_**k**_∥_ ± 2α**Δk**_**x**__, with α ≥ 1 and an integer, through the light-matter coupling to excitons. However, this coupling fades exponentially with increasing α with the effective coupling at α = 1 being the strongest.

As a result of this effective coupling, for a given *k*_*x*_ (with *k*_*y*_ = 0 fixed) in addition to the original polariton bands *E*_±_ (*k*_*x*_, *k*_*y*_ = 0), new bands at *E*_±_ (*k*_*x*_ ± Δ*k*_*x*_, *k*_*y*_ = 0) emerge. These bands are clearly visible in Figure 2g. Importantly, these band replicas also strongly interact with the original polariton bands near **k**_∥_ → **0** and leads to the formation of flat bands. We anticipate that this nondispersive character of the polariton flat bands will open new opportunities for inducing exotic physical phenomena, as observed in 2D moiré heterostructures.^27,36–38^ Specifically, the emergence of flat bands implies a substantial enhancement of the density of states at **k**_∥_ → **0**, which may facilitate the formation of polariton condensates at room temperature. At the same time, these flat bands are also expected to profoundly impact the transport properties of exciton-polaritons. Since the associated group velocities are zero for these flat-bands we expect the formation of coherent nonpropagating polariton density in the direction of the material tilt. While these interesting aspects lie beyond the scope of the present work, LMME-induced polariton condensate formation as well as the LMME-modified polariton transport will be studied in our future work.

Figure 2f shows the polariton dispersion along *k*_*y*_ (orthogonal to the tilt direction) at *k*_*x*_ = 0, featuring multiple energy-shifted replicas of the original polaritonic bands. All polariton bands near *k*_*y*_ = 0 exhibit finite curvature, indicating a finite effective mass. This illustrates that tilting a (isotropic) material along one direction leads to an anisotropic flatband, which is expected to lead to anisotropic polariton transport and anisotropic localization.

Figure 2h display the 2D cut of the polariton dispersion at a fixed energy *E* = 3 eV. In comparison to the untilted scenario, presented in Figure 2d, Figure 2h shows (at least) three circles with two displaced along the *x* direction which closely resemble a Rashba-Dresselhaus-like splitting seen in polaritonic spin-Hall effect.^1^ Notably, unlike the spin-Hall effect, the original polariton band does not disappear in the LMME. The displacement between the band replicas shown in Figure 2g–h depends on the angle of tilt θ and is given by $\Delta k_{x}=k_{z}\sin\left(\theta \right)=\frac{m_{z}\pi }{L_{z}}\sin\left(\theta \right)$ . Therefore, a larger displacement in reciprocal space can be achieved at a smaller tilt angle when using a higher cavity mode *m*_*z*_. In analogy to the Rashba-Dresselhaus Hamiltonian,^1^ these (uncoupled) side bands can be *crudely* described by

12 $${Ĥ}_{\pm }=-\frac{\hbar^{2}}{2m_{\mathrm{eff}}}\nabla_{∥}^{2}\pm 2ik_{z}\sin\left(\theta \right)\frac{\hbar }{m_{\mathrm{eff}}}\frac{\partial }{\partial x}$$

where *m*_eff_ is the effective mass extracted from the curvature of the polaritonic dispersion. We emphasize, however, that unlike in the polariton spin-Hall effect, these ${Ĥ}_{\pm }$ do not correspond to two circularly polarized light modes; in our case, both side bands are elliptically polarized.

In Figure 3, we illustrate how varying the tilt angle and stacking multiple material layers influence the polariton dispersion and the resulting LMME. As illustrated in Figure 3a, the angle of tilt θ increases monotonically (and almost linearly) with Δ*k*_*x*_. This is expected as at small θ as $\Delta k_{x}\approx \frac{m_{z}\pi }{L_{z}}\theta$ . This monotonic increase in Δ*k*_*x*_ can also be seen in Figure 3b–d, which shows a 2D cut of the polariton dispersion at θ = 3.58, 5.98, and 8.38°, respectively. At small θ, more than two displaced polariton bands appear, alongside the original polariton band, seen in Figure 3b. This occurs because, at smaller displacements of Δ*k*_*x*_, the energy difference between photonic modes for α = 2 (i.e., between *Â*_**k**_∥__ and *Â*_**k**_∥_ ± **4Δk**_**x**__) is sufficiently small to enable appreciable hybridization, despite the significantly weaker effective couplings. Consequently, at slightly higher angles of tilt in Figure 3c–d only two displaced polaritons bands appear. Figure 3f–h illustrate how stacking multiple material layers influence the polariton dispersion under a constant tilt (θ = 5.98°) for 5, 10, and 40 layers, respectively. To provide an analytical understanding of how polaritonic dispersion are modified in a multilayered setup, consider the light-matter Hamiltonian rewritten using a bright layer formalism presented in our recent works.^31,39^ In this formalism a multilayered material is described using an effective (single) bright layer which couples to quantized radiation. The effective bright-layer Hamiltonian $\left({Ĥ}_{\mathrm{AX}}\to {Ĥ}_{\mathrm{AX}}^{B}\right)$ is written as (with details provided in the SI)­

13 $${Ĥ}_{\mathrm{AX}}^{B}=\sum_{k_{∥}}{Â}_{k_{∥}}^{†}{Â}_{k_{∥}}\omega_{k}+\sum_{k_{∥}}\left({\mathrm{X̂}}_{k_{∥},B}^{†}{\mathrm{X̂}}_{k_{∥},B}\right)\epsilon_{k_{∥}}+\Omega_{0}\sum_{n_{x},k_{y}}\sqrt{N_{n_{x}}}\left({\mathrm{X̂}}_{n_{x},k_{y},B}^{†}{Â}_{n_{x},k_{y}}+{\mathrm{X̂}}_{n_{x},k_{y},B}{Â}_{n_{x},k_{y}}^{†}\right)$$

where ${\mathrm{X̂}}_{n_{x},k_{y},B}^{†}=\frac{1}{\sqrt{N_{n_{x}}}}\sum_{n_{z}}\sin\left(k_{z}z_{m}\right){\mathrm{X̂}}_{n_{x},k_{y},n_{z}}$ with $N_{n_{x}}=\sum_{n_{z}}\sin^{2}\left(k_{z}z_{m}\right)$ a normalization constant. Since $N_{n_{x}}$ is approximately proportional to *N*_*z*_, $\sqrt{N_{n_{x}}}\Omega_{0}$ is almost a constant when varying the number of layers in Figure 3f–h ensuring a fair comparison. Note that all numerical results presented here employ a full description of the multilayer material and its coupling to the quantized radiation (as described in eq 10), involving no additional approximations.

**Figure 3:**
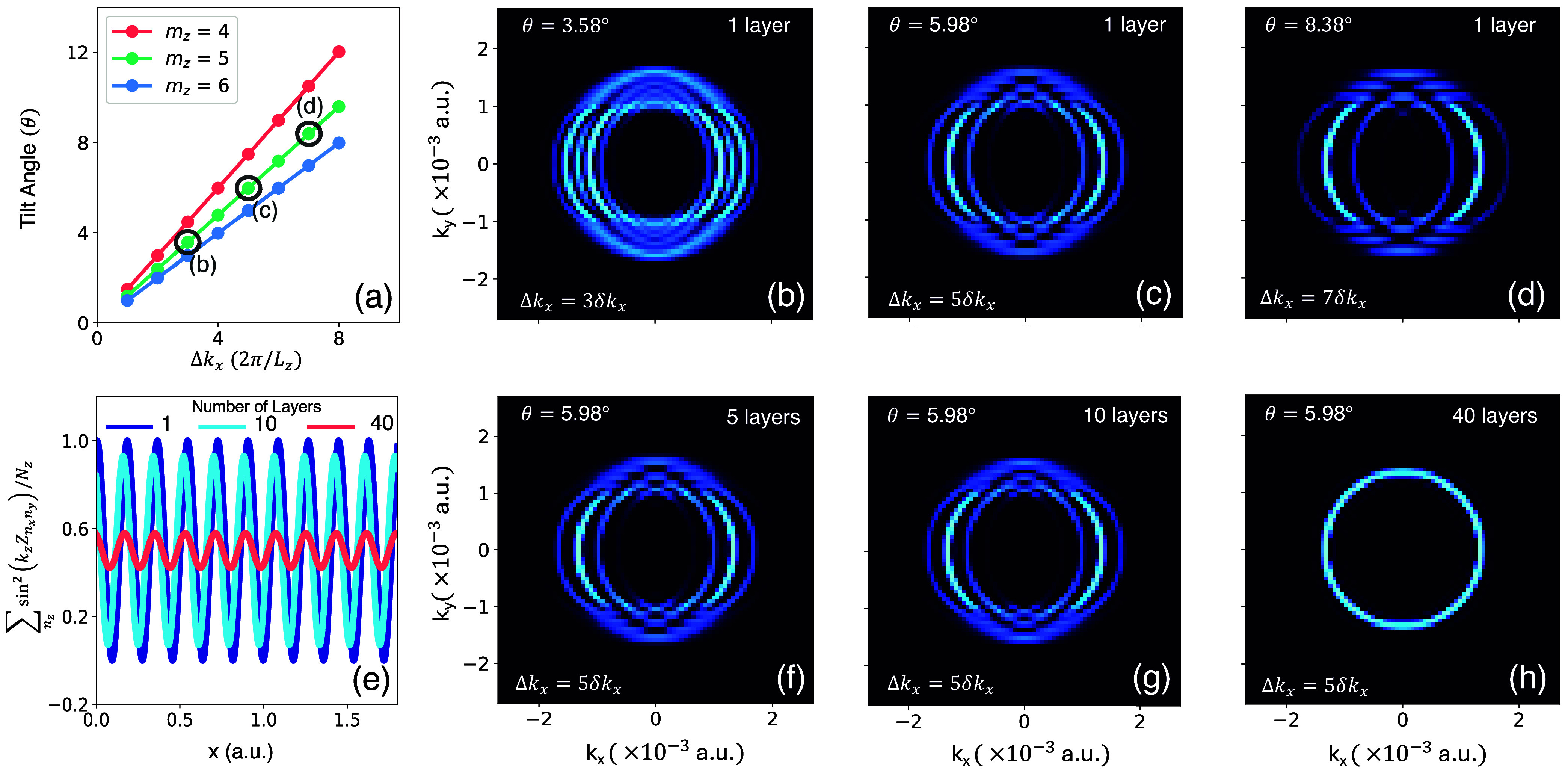
Tilt-induced separation of side bands and their dependence on material thickness. (a) Relation between tilt angle θ and Δ*k*_*x*_ for the model system. (b–d) Two dimensional band structures for tilts of θ = 3.58, 5.98, 8.38° corresponding to Δ*k*_*x*_, where δ*k*_*z*_ = 2π/*L*_*x*_ and *p* ∈ {3,5,7}, respectively for a single layer. (e) Normalized spatially varying (bright-layer) light matter coupling at *n*_*z*_ = 1, 10, 40. (f–h) Two dimensional band structures of tilt θ = 5.98° corresponding to Δ*k*_*x*_ = 5δ*k*_*x*_ for *n*_*z*_ = 5, 10, 40, respectively. We use *N*_*x*_ = *N*_*y*_ = 8001.

Figure 3f–g presents the polariton dispersion when stacking 5 and 10 layers, respectively. These polariton dispersions are nearly identical to the single layer result in Figure 3c with the same angle of tilt. This is because, at *N*_*z*_ = 5 or 10, the thickness of the material remains much smaller than the distance between the mirrors (10*a*_*z*_ = 300 Å≪ *L*_*z*_) such that it can be effectively regarded as single layer material. Mathematically, $N_{n_{x}}\approx N_{z}\sin^{2}\left(k_{z}z_{n_{x},0}\right)$ such that eq 13 reduces to the single layer version of the ${Ĥ}_{\mathrm{AX}}$ in eq 10, i.e., ${Ĥ}_{\mathrm{AX}}^{B}\to {Ĥ}_{\mathrm{AX}}\left(N_{z}=1\right)$ for *N*_*z*_ *a*_*z*_ ≪ *L*_*z*_. This suggests that LMME is not limited to a single layer material but it can be observed as long as the material thickness is much smaller than the thickness of the optical cavity.

Figure 3h presents the polaritonic dispersion when stacking 40 layers which corresponds to a material thickness of 1200 Å. At this thickness, the LMME fully disappears, and the polaritonic dispersion resembles that of the untilted material (see Figure 2d). This behavior can be understood by inspecting $N_{n_{x}}/N_{z}$ , which characterizes the spatially varying light-matter couplings under material tilt. Figure 3e plots $N_{n_{x}}/N_{z}$ as a function of *x*. In the single-layer case (*N*_*z*_ = 1), $\frac{N_{n_{x}}}{N_{z}}=\sin^{2}\left(k_{z}z_{n_{x},0}\right)$ , which results in strong spatial oscillations (dark blue solid line in Figure 3e) that give rise to the LMME. Importantly, for *N*_*z*_ = 10, $N_{n_{x}}/N_{z}$ remains oscillatory and closely matches the single-layer case. At *N*_*z*_ = 40, the oscillation is greatly reduced and to a good approximation $N_{n_{x}}/N_{z}$ could be regarded as a constant. Note that replacing $N_{n_{x}}$ to a spatially independent constant reduces the Hamiltonian in eq 13 to the untilted scenario. This explains why the polariton dispersion in Figure 3h resembles the untilted scenario in Figure 2d.

Overall, LMME induced by tilting a material inside an optical cavity can unlock exotic new phenomena, including the formation of momentum-displaced polariton bands and anisotropic flat bands. Below, we illustrate one exciting application of LMME: coherent frequency conversion, which is of enormous importance in quantum information processing.^40–43^

Figure 4 illustrates the applicability of using LMME for performing coherent frequency conversion. This process is schematically illustrated in Figure 4a. We prepare an initial superposition of two single-photon modes with relative phase ϕ written as

14 $$\left|\Psi \left(0\right)\right\rangle =\frac{1}{\sqrt{2}}\left[{Â}_{k_{∥}}^{†}+e^{i\phi }{Â}_{k_{∥}^{\prime }}^{†}\right]\left|\mathrm{0̅}\right\rangle$$

**Figure 4:**
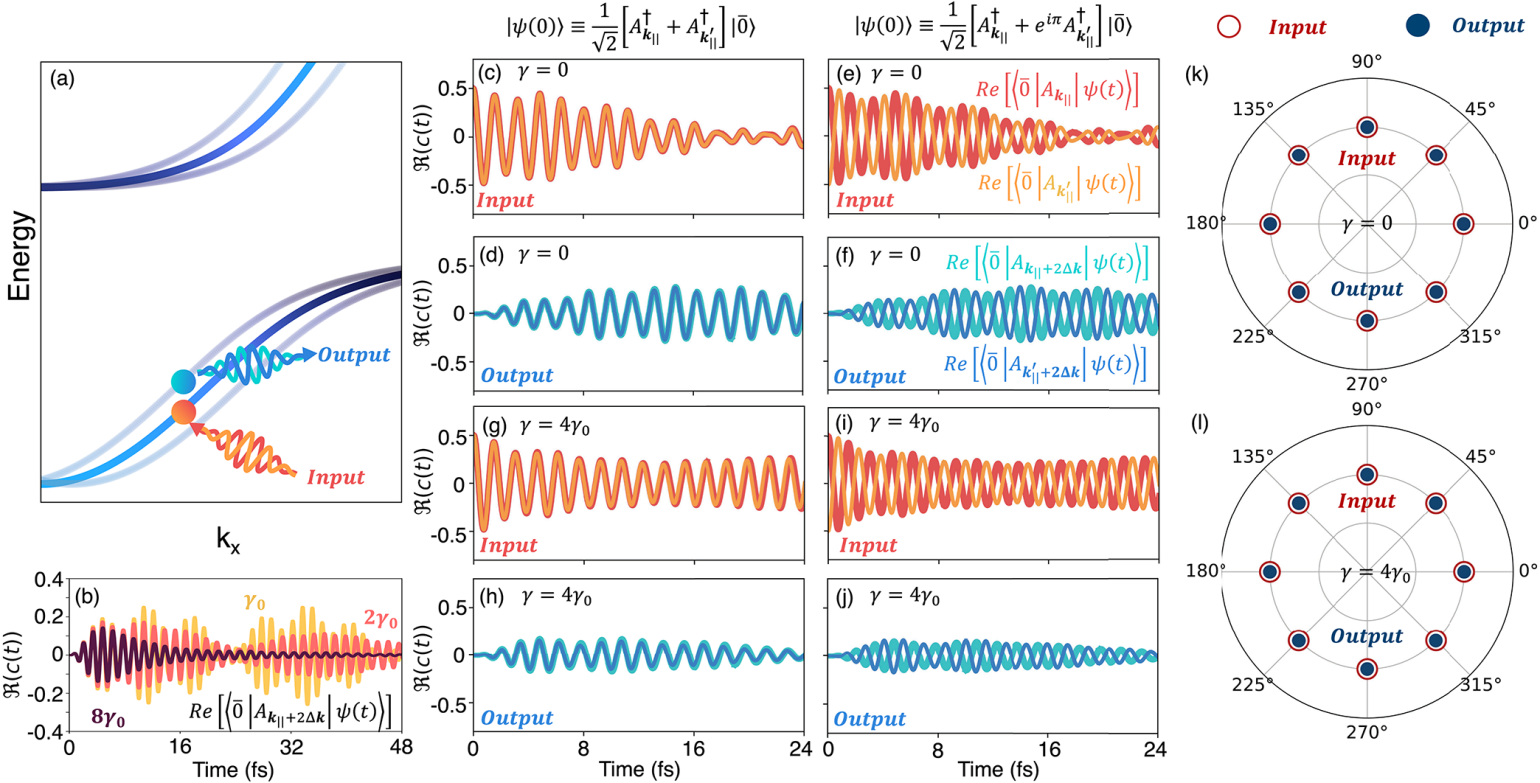
Coherent frequency conversion. (a) Band structure depicting an input coherent superposition $\frac{1}{\sqrt{2}}\left[{Â}_{k_{∥}}^{†}+e^{i\phi }{Â}_{k_{∥}^{\prime }}^{†}\right]\left|\mathrm{0̅}\right\rangle$ , and an output coherent superposition of states $\frac{1}{\sqrt{2}}\left[{Â}_{k_{∥}+2\Delta k}^{†}+e^{i\phi }{Â}_{k_{∥}^{\prime }+2\Delta k}^{†}\right]\left|\mathrm{0̅}\right\rangle$ , for the material at angle of tilt θ = 5.98° corresponding to **Δk** = 5δ*k*_*x*_. (b) Real component of state *Â*_**k**_∥__^†^|0̅⟩ with |ψ­(*t*)⟩ over 48 fs, with phonon couplings equal to γ_0_, 2γ_0_, 8γ_0_, where γ_0_ = 7.3067 × 10^–5^ a.u. (c–d) The input and output superposition over 24 fs, without phonon coupling or an initial phase difference between states *Â*_**k**_∥__^†^|0̅⟩ and *Â*_**k**_∥_^′^_^†^| 0̅⟩. (e–f) The input and output superposition over 24 fs, without phonon coupling and an initial phase difference of ϕ = π between states *Â*_**k**_∥__^†^|0̅⟩ and *Â*_**k**_∥_^′^_^†^|0̅⟩. (g–j) Mimic the structure of (c–f), differing as the phonon coupling γ = 4γ_0_. (k–l) Polar plots displaying the initial phase difference (after 0.5 fs) of the input and output in 45° intervals, for γ = 0 (k) and γ = 4γ_0_ (l).

Here we choose $k_{∥}=20\frac{2\pi }{L_{z}}\mathrm{x⃗}+0\mathrm{y⃗}$ and $k_{∥}^{\prime }=21\frac{2\pi }{L_{z}}\mathrm{x⃗}+0\mathrm{y⃗}$ for preparing our initial condition which corresponds to a photon frequency of ω_**k**_∥__ = 2.798 eV and ω_**k**_∥_^′^_ = 2.819 eV, respectively. Given this initial state, we detect two photon at **k**_∥_ + 2**Δk** and **k**_∥_^′^ + 2**Δk** (corresponds to a photon frequency of ω_**k**_∥__ = 3.046 eV and ω_**k**_∥_^′^_ = 3.075 eV, respectively for a tilt angle θ = 5.98° with *N*_*z*_ = 1) and measure their phase difference. Specifically, we compute ⟨0̅|*Â*_**k**_∥_ + 2**Δk**_ |Ψ­(*t*)⟩ and ⟨0̅|*Â*_**k**_∥_^′^ + 2**Δk**_ |Ψ­(*t*)⟩.

Figure 4c–h present numerical results demonstrating coherent frequency conversion enabled by tilting the material inside an optical cavity. In Figure 4c–d, we set the initial relative phase ϕ = 0 and simulate the dynamics in the absence of any phonons. Figure 4c plots ⟨0̅|*Â*_**k**_∥__|Ψ­(*t*)⟩ (red solid line) and ⟨0̅|*Â*_**k**_∥_^′^_|Ψ­(*t*)⟩ (orange solid line) as functions of time, both of which lie on top of each other, as expected for ϕ = 0. The outputs, ⟨0̅|*Â*_**k**_∥_ + 2Δ**k**_|Ψ­(*t*)⟩ and ⟨0̅|*Â*_**k**_∥_^′^ + 2Δ**k**_|Ψ­(*t*)⟩ (cyan and blue solid lines in Figure 4d), also lie on top of each other, indicating that the output phase difference (at *t* → 0) is 0°.

In Figure 4e–f, we set the initial relative phase to ϕ = π and simulate the dynamics in the absence of phonons. Consequently, Figure 4e shows that ⟨0̅|*Â*_**k**_∥__|Ψ­(*t*)⟩ (red solid line) and ⟨0̅|*Â*_**k**_∥_^′^_|Ψ­(*t*)⟩ (orange solid line) oscillate out of phase by ϕ = π. Importantly, Figure 4f shows that ⟨0̅|*Â*_**k**_∥_ + 2Δ**k**_|Ψ­(*t*)⟩ and ⟨0̅|*Â*_**k**_∥_^′^ + 2Δ**k**_|Ψ­(*t*)⟩ also oscillate out of phase, mirroring the input phase difference by π. These results illustrate that a superposition of two photons at **k**_∥_ and **k**_∥_^′^ can be converted to a superposition of two photons at **k**_∥_ + 2Δ**k** and **k**_∥_^′^ + 2Δ**k** which carry the same relative phase encoded in the initial state. Figure 4k, shows that this scheme is broadly applicable, where we have plotted the relative initial phase difference between the input and output phase that is evaluated as $\phi_{\mathrm{output}}=\lim_{t\to 0}\arg\left[\frac{\left\langle \mathrm{0̅}\right|{Â}_{k_{∥}+2\Delta k}\left|\Psi \left(t\right)\right\rangle }{\left\langle \mathrm{0̅}\right|{Â}_{k_{∥}^{\prime }+2\Delta k}\left|\Psi \left(t\right)\right\rangle }\right]$ with more numerical details provided in the SI.

To assess the feasibility of performing coherent frequency conversion in the presence of phonon induced disorder, we simulate the following 1D generalized Holstein-Tavis-Cummings Hamiltonian written as

15 $${Ĥ}_{\mathrm{HTC}}={Ĥ}_{\mathrm{AX}}^{k_{y}=0}+\sum_{m}{\mathrm{b̂}}_{m}^{†}{\mathrm{b̂}}_{m}\omega_{b}+\frac{\gamma }{\sqrt{2\omega_{b}}}\sum_{m}{\mathrm{X̂}}_{m,0}^{†}{\mathrm{X̂}}_{m,0}\left({\mathrm{b̂}}_{m}^{†}+{\mathrm{b̂}}_{m}\right)$$

where *b̂*_**m**_^†^ creates a phonon of frequency ω_b_ at a site **m** ∈ (*n*_*x*_, *n*_*z*_) ≡ (*n*_*x*_, 0) (with *N*_*z*_ = 1) and γ is the exciton–phonon coupling strength. Note in this simple 1D model we ignore Fröhlich scattering in the *k*_*y*_ direction where ${Ĥ}_{\mathrm{AX}}^{k_{y}=0}$ is coupled to ${Ĥ}_{\mathrm{AX}}^{k_{y}\neq 0}$ . We make this *simplifying* approximation following our recent work where we find that phonon induced Fröhlich scattering of polaritons is negligible.^25^ We utilize a mixed quantum-classical approach, namely mean-field ehrenfest,^33,44–46^ to propagate *Ĥ*_HTC_ (see details in SI). In Figure 4g–j we illustrate effect of the exciton–phonon couplings on the coherent frequency conversion via LMME. We find that the inputs and outputs remains phase-locked, similar to the phonon-free case presented in Figure 4c–f. Note that this is despite using a relatively strong exciton–phonon coupling (γ = 4γ_0_ with γ_0_ ≈ 7.3067 × 10^–5^ au); by comparison, typical polycyclic aromatic hydrocarbons (and their derivatives^47^) have γ ≈ γ_0_. Figure 4l illustrates that the relative phases between input and output photons remain the same even in the presence of strong phonon interactions. Note that the Huang–Rhys (HR) factor *S* is connected to the reorganization energy λ = γ^2^/(2ω^2^) through *S* = λ/(ℏω). For our simulations involving phonon coupling, we have considered a single phonon mode with frequency ω = 360 cm^–1^, for which the corresponding HR factor associated with the reference coupling γ_0_ is *S* = 0.605 which corresponds to a Debye-Weller factor *W* = *e* ^–S^ ≈ 0.546. As expected, an increase in exciton–phonon coupling leads to more decoherence marked with increased decay in oscillation in Figure 4b. Importantly, decoherence becomes substantial only at extreme exciton–phonon coupling (γ = 8γ_0_). This is expected as we are performing frequency conversion far away from the excitonic transition at 3.2 eV such that the effective polariton-phonon couplings are substantially suppressed.^3,48^

## Conclusions

In this work, we introduce and theoretically characterize the light-matter moiré effect (LMME) that emerges when an isotropic 2D material is tilted inside a 3D Fabry-Pérot cavity. Unlike conventional moiré phenomena in twisted multilayer systems, LMME arises purely from geometric modulation of the light-matter coupling. This produces momentum-displaced replicas of the polariton dispersion, in close analogy to the Rashba and Dresselhaus spin–orbit couplings, and enables the formation of anisotropic flat bands near the Brillouin-zone center.

We demonstrate that LMME can be used to perform coherent frequency conversion, where the relative phase encoded in an initial superposition of two photons is transferred to a superposition of two photons at a different frequency with a frequency shift that is determined by the angle of tilt. Importantly,we find (using an approximate 1D model) that the phase information is preserved in even in the presence of substantial exciton–phonon interactions, suggesting its robust nature and its potential application in quantum information processing.

Beyond its fundamental interest, LMME offers a versatile platform for engineering polariton band structures and tailoring light-matter interactions. The ability to generate tunable flat bands and perform phase-preserving frequency conversion through cavity-material architecture opens new avenues for engineering polariton-based quantum devices, directional transport, and efficient room-temperature polariton condensation.

## Supplementary Material



## Notes

Description of the quantized electric fields; real-reciprocal space representation of the light-matter interaction Hamiltonian; light-matter Hamiltonian in the reciprocal space and in effective bright-layer representation; details of the quantum dynamical approach; details of evaluating time-independent phase difference (PDF)
