# Supplementary material for: Tilted Material in an Optical Cavity: Light-Matter Moiré Effect and Coherent Frequency Conversion
Source: ACS Photonics. 2025 Oct 28;12(12):6911–9. doi: 10.1021/acsphotonics.5c02118 (PMC12716130; doi:10.1021/acsphotonics.5c02118)
Supplement: Supplementary file 1 [file ph5c02118_si_001.pdf]

Tilted Material in an Optical Cavity: Light-Matter Moiré Effect and Coherent Frequency Conversion

Arshath Manjalingal[†], Saeed Rahmanian Koshkaki[†*], Logan Blackham, Arkajit Mandal[*]

October 12, 2025

Contents

S1 Quantized Electric Fields **S2**

S2 Real–Reciprocal Space Representation of the Light–Matter Interaction Hamiltonian **S2**

S3 Light-Matter Hamiltonian in the Reciprocal Space and in Effective Bright-Layer Representation **S3**

S4 Quantum Dynamical Approach **S4**

S5 Evaluating Time-Independent Phase Difference **S5**

[*]Corresponding Authors: rahmanian@tamu.edu, mandal@tamu, [†] Equal contribution

S1 Quantized Electric Fields

For an optical cavity formed by two perfect mirrors and filled with an isotropic dielectric medium, the solution to Maxwell's equations gives rise to two polarization modes of the electric field, denoted by $\bar{\mathbf{E}}^{s}_{\mathbf{k}}(\mathbf{R_n})\hat{a}_{\mathbf{k}_\parallel}$ and $\bar{\mathbf{E}}^{p}_{\mathbf{k}}(\mathbf{R_n})\hat{b}_{\mathbf{k}_\parallel}$ (with their normalized counterpart $\mathbf{E}^{s}_{\mathbf{k}}(\mathbf{R_n})$ and $\mathbf{E}^{s}_{\mathbf{k}}(\mathbf{R_n})$), which are defined as transverse electric (TE) and transverse magnetic (TM) modes [1], respectively, and represented as

$$\bar{\mathbf{E}}^{s}_{\mathbf{k}}(\mathbf{R_n}) = g\mathbf{E}^{s}_{\mathbf{k}}(\mathbf{R_n}) = g\sin(k_z z_{\mathbf{n}})\{\vec{e}_\parallel \times \vec{z}\}e^{i\mathbf{k}_\parallel\cdot\mathbf{R_n}} \tag{S1}$$

$$\bar{\mathbf{E}}^{p}_{\mathbf{k}}(\mathbf{R_n}) = g\mathbf{E}^{p}_{\mathbf{k}}(\mathbf{R_n}) = g\left[\frac{c|\mathbf{k}_\parallel|}{\omega_{\mathbf{k}}}\cos(k_z z_n)\vec{z} - i\frac{ck_z}{\omega_{\mathbf{k}}}\sin(k_z z_n)\vec{e}_\parallel\right]e^{i\mathbf{k}_\parallel\cdot\mathbf{R_n}}, \tag{S2}$$

Here, $g \propto \frac{1}{\sqrt{V}}$ determines the strength of the quantized radiation. The total electric field $\mathbf{E}$ can be written as,

$$\vec{\mathbf{E}} = \bar{\mathbf{E}}^{s}_{\mathbf{k}}(\mathbf{R_n}) + \bar{\mathbf{E}}^{p}_{\mathbf{k}}(\mathbf{R_n}) = g[\mathbf{E}^{s}_{\mathbf{k}}(\mathbf{R_n}) + \mathbf{E}^{s}_{\mathbf{k}}(\mathbf{R_n})] = E_x\vec{x} + E_y\vec{y} + E_z\vec{z} \tag{S3}$$

$$E_x = g\left(\frac{k_y}{|\mathbf{k}_\parallel|}\hat{a}_{\mathbf{k}_\parallel} - i\frac{ck_z}{\omega_{\mathbf{k}}}\frac{k_x}{|\mathbf{k}_\parallel|}\hat{b}_{\mathbf{k}_\parallel}\right)\sin(k_z z_{\mathbf{n}})e^{i\mathbf{k}_\parallel\cdot\mathbf{R_n}} + h.c. \tag{S4}$$

$$E_y = g\left(-\frac{k_x}{|\mathbf{k}_\parallel|}\hat{a}_{\mathbf{k}_\parallel} - i\frac{ck_z}{\omega_{\mathbf{k}}}\frac{k_y}{|\mathbf{k}_\parallel|}\hat{b}_{\mathbf{k}_\parallel}\right)\sin(k_z z_{\mathbf{n}})e^{i\mathbf{k}_\parallel\cdot\mathbf{R_n}} + h.c. \tag{S5}$$

$$E_z = g\left(\frac{c|\mathbf{k}_\parallel|}{\omega_{\mathbf{k}}}\hat{b}_{\mathbf{k}_\parallel}\right)\cos(k_z z_{\mathbf{n}})e^{i\mathbf{k}_\parallel\cdot\mathbf{R_n}} + h.c. \tag{S6}$$

Here, E_x, E_y, and E_z denote the components of the quantized electric field along the $\vec{x}$-, $\vec{y}$-, and $\vec{z}$-axes, respectively. The wavevector $\mathbf{k} = (\mathbf{k}_\parallel, k_z)$ consists of the in-plane component $\mathbf{k}_\parallel = (k_x, k_y)$ and the out-of-plane component k_z, with $|\mathbf{k}_\parallel| = \sqrt{k_x^2 + k_y^2}$ denoting the magnitude of the in-plane wavevector. The operators $\hat{a}_{\mathbf{k}}$ and $\hat{b}_{\mathbf{k}}$ represent the annihilation operators for the two orthogonal polarization modes of the electric field, typically corresponding to the TE (s-polarized) and TM (p-polarized) modes, respectively. The quantity $\omega_{\mathbf{k}}$ is the frequency of the cavity photon mode with wavevector $\mathbf{k}$, and c denotes the speed of light in the dielectric medium. The position vector of the lattice site indexed by $\mathbf{n} = (n_x, n_y, n_z)$ is given by $\mathbf{R_n} = (x_{\mathbf{n}}, y_{\mathbf{n}}, z_{\mathbf{n}})$.

S2 Real–Reciprocal Space Representation of the Light–Matter Interaction Hamiltonian

The exciton-cavity interaction Hamiltonian ($\hat{H}_{\text{int}}$) for this sytem can be is written as,

$$\hat{H}_{\text{int}} = \frac{g}{\sqrt{N}}\sum_{\mathbf{n},\mathbf{k},j}\sqrt{\omega_{\mathbf{k}}}\left[\hat{\boldsymbol{\mu}}_j\cdot\left(\mathbf{E}^{s}_{\mathbf{k}}(\mathbf{R_n})\hat{a}_{\mathbf{k}} + \mathbf{E}^{p}_{\mathbf{k}}(\mathbf{R_n})\hat{b}_{\mathbf{k}}\right) + h.c.\right]. \tag{S7}$$

In our model, every site accommodates two degenerate excited states, which gives rise to two transition dipole moments, $\hat{\mu}_x$ and $\hat{\mu}_y$, aligned in the $\vec{x}$ and $\vec{y}$ axes, respectively. Consequently, the transition dipole operator is defined by $\hat{\boldsymbol{\mu}}_1 = \hat{\boldsymbol{\mu}}^{x}_{\mathbf{n}} = \mu_x\left(\hat{X}_{\mathbf{n}} + \hat{X}^{\dagger}_{\mathbf{n}}\right)\vec{x}$ and $\hat{\boldsymbol{\mu}}_2 = \hat{\boldsymbol{\mu}}^{y}_{\mathbf{n}} = \mu_y\left(\hat{Y}_{\mathbf{n}} + \hat{Y}^{\dagger}_{\mathbf{n}}\right)\vec{y}$, here $\hat{X}^{\dagger}_{\mathbf{n}}$ and $\hat{Y}^{\dagger}_{\mathbf{n}}$ are the exciton creation operators. In this work we consider an isotropic material, i.e. $\mu_x = \mu_y = \mu$. Note that the E_z contribution is vanishingly small (for the tilt angles considered in this work), thus it is neglected. We write the light-matter interaction as

$$\begin{aligned}\hat{H}_{\text{int}} = \frac{\mu g}{\sqrt{N}}\sum_{\mathbf{n},\mathbf{k}_\parallel}\sqrt{\omega_{\mathbf{k}}}\Bigg(&\left(\frac{k_y}{|\mathbf{k}_\parallel|}\hat{a}^{\dagger}_{\mathbf{k}_\parallel}e^{-i\mathbf{k}_\parallel\cdot\mathbf{R_n}} + i\frac{ck_z}{\omega_{\mathbf{k}}}\frac{k_x}{|\mathbf{k}_\parallel|}\hat{b}^{\dagger}_{\mathbf{k}_\parallel}e^{-i\mathbf{k}_\parallel\cdot\mathbf{R_n}} + h.c.\right)\left(\hat{X}_{\mathbf{n}} + \hat{X}^{\dagger}_{\mathbf{n}}\right) - \\ &\left(\frac{k_x}{|\mathbf{k}_\parallel|}\hat{a}^{\dagger}_{\mathbf{k}_\parallel}e^{-i\mathbf{k}_\parallel\cdot\mathbf{R_n}} - i\frac{ck_z}{\omega_{\mathbf{k}}}\frac{k_y}{|\mathbf{k}_\parallel|}\hat{b}^{\dagger}_{\mathbf{k}_\parallel}e^{-i\mathbf{k}_\parallel\cdot\mathbf{R_n}} + h.c.\right)\left(\hat{Y}_{\mathbf{n}} + \hat{Y}^{\dagger}_{\mathbf{n}}\right)\Bigg)\sin(k_z z_{\mathbf{n}}).\end{aligned} \tag{S8}$$

We introduce the elliptically polarized photonic operators $\hat{A}_{\mathbf{k}_\parallel}$ and $\hat{B}_{\mathbf{k}_\parallel}$ written as

$$\begin{aligned}\hat{A}_{\mathbf{k}_\parallel} &= \frac{1}{\sqrt{\mathcal{S}_x(\mathbf{k})}}\left(k_y\hat{a}_{\mathbf{k}_\parallel} + i\frac{ck_z}{\omega_k}k_x\hat{b}_{\mathbf{k}_\parallel}\right)\\ \hat{B}_{\mathbf{k}_\parallel} &= \frac{1}{\sqrt{\mathcal{S}_y(\mathbf{k})}}\left(k_x\hat{a}_{\mathbf{k}_\parallel} - i\frac{ck_z}{\omega_k}k_y\hat{b}_{\mathbf{k}_\parallel}\right),\end{aligned} \tag{S9}$$

where $\mathcal{S}_x(\mathbf{k}) = 1 - \frac{c^2k_x^2}{\omega_{\mathbf{k}}^2}$ and $\mathcal{S}_y(\mathbf{k}) = 1 - \frac{c^2k_y^2}{\omega_{\mathbf{k}}^2}$ are normalization constants chosen so that the operators satisfy the canonical bosonic commutation relations. By substituting S9 in S8 we obtain the following expression for the light-matter interaction as

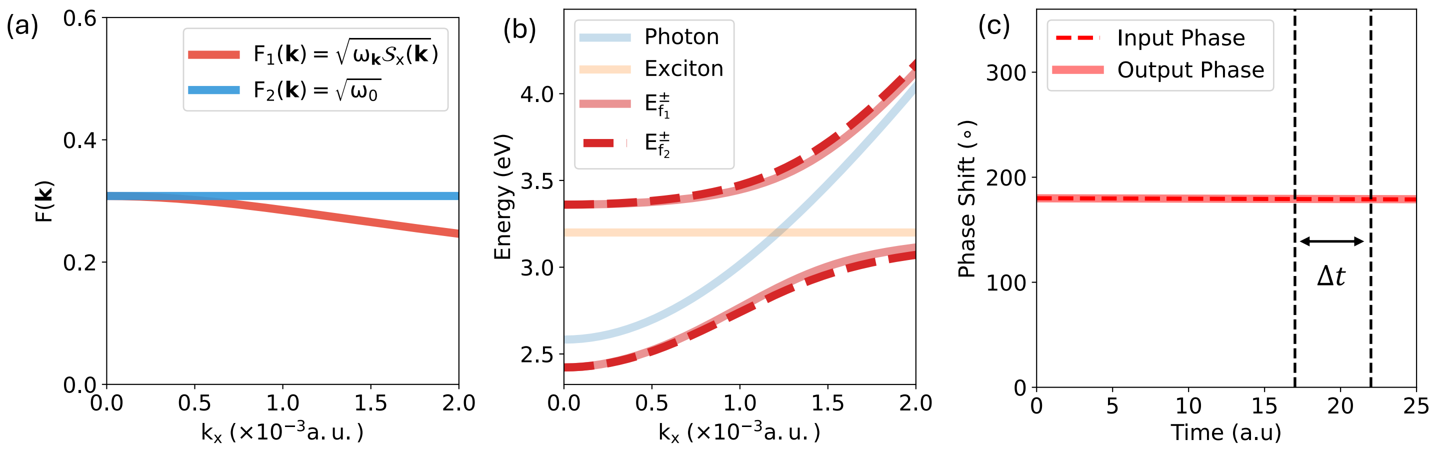

Figure S1: **Comparison of Light–Matter Coupling With and Without the Longitudinal Mode Approximation** (a) Comparison of $F_1(\mathbf{k}) = \sqrt{\omega_\mathbf{k} \mathcal{S}_x(\mathbf{k})}$ and the approximated constant $F_2(\mathbf{k}) = \sqrt{\omega_0}$ as functions of k_x, highlighting the validity of the approximation $|\mathbf{k}_\parallel| \ll k_z$, where $\omega_0 = \sqrt{\omega_\mathbf{k} \mathcal{S}_x(\mathbf{k})} \approx \sqrt{\omega_\mathbf{k} \mathcal{S}_y(\mathbf{k})}$. This approximation is justified within the selected regime, only the lowest 0.8% of the photonic modes were considered. (b) Dispersion of the upper and lower polariton branches computed using both $F_1(\mathbf{k})$ and the approximated $F_2(\mathbf{k})$. The close agreement in the small-k_x regime confirms the validity of the approximation. Photon and exciton bands are indicated by arrows. **Calculating the Phase Shift from Input and Output** (c) Phase shifts ($\phi_{\text{output}}(t)$ and $\phi_{\text{input}}(t)$) plotted as a function of time. The effective phase difference is obtained by evaluating over the interval Δt.

$$\hat{H}_{\text{int}} = \frac{\mu g}{\sqrt{N}} \sum_{\mathbf{n},\mathbf{k}} \sin(k_z z_\mathbf{n}) \sqrt{\omega_\mathbf{k}} \left(\sqrt{\mathcal{S}_x(\mathbf{k})} \left(\hat{A}_\mathbf{k} e^{i\mathbf{k}_\parallel \cdot \mathbf{R_n}} + \hat{A}^\dagger_\mathbf{k} e^{-i\mathbf{k}_\parallel \cdot \mathbf{R_n}} \right) \left(\hat{X}_\mathbf{n} + \hat{X}^\dagger_\mathbf{n} \right) - \sqrt{\mathcal{S}_y(\mathbf{k})} \left(\hat{B}_\mathbf{k} e^{i\mathbf{k}_\parallel \cdot \mathbf{R_n}} + \hat{B}^\dagger_\mathbf{k} e^{-i\mathbf{k}_\parallel \cdot \mathbf{R_n}} \right) \left(\hat{Y}_\mathbf{n} + \hat{Y}^\dagger_\mathbf{n} \right) \right). \tag{S10}$$

Noting that the relevant dynamics (and dispersion) occur for $|\mathbf{k}_\parallel| \ll k_z$ for which we can make the approximation $\sqrt{\omega_0} \approx \sqrt{\omega_k \mathcal{S}_x(\mathbf{k})} = \sqrt{\omega_k \mathcal{S}_y(\mathbf{k})}$. Fig. S1b demonstrates applicability of this approximation. Fig. S1b shows that $E^{\pm}_{f_1}$ (polaritonic band without the approximation) are close to the polaritonic bands $E^{\pm}_{f_2}$ obtained using this approximation with $\sqrt{\omega_0}\mu g = 0.35$ eV (we use $N_z = 1$). The small deviation between the two curves are inconsequential for the results presented in this work. Further, we employ the rotating wave approximation and perform a partial Fourier transform $\hat{X}_{n_x,k_y} = \frac{1}{\sqrt{N_y}} \sum_{n_y} \hat{X}_{1,n_x,n_y} e^{-ik_y n_y a_y}$, $\quad \hat{A}_{n_x,k_y} = \frac{1}{\sqrt{N_y}} \sum_{k_x} \hat{A}_{k_x,k_y} e^{ik_x n_x a_x}$ and obtain the light-matter interaction Hamiltonian in the real-reciprocal representation as

$$H_{\text{int}} = \frac{\sqrt{\omega_0}\mu g}{\sqrt{N_z}} \sum_{\mathbf{m},k_y} \left([\hat{X}^\dagger_{\mathbf{m},k_y} \hat{A}_{\mathbf{m},k_y} + \hat{X}_{\mathbf{m},k_y} \hat{A}^\dagger_{\mathbf{m},k_y}] - [\hat{Y}^\dagger_{\mathbf{m},k_y} \hat{B}_{\mathbf{m},k_y} + \hat{Y}_{\mathbf{m},k_y} \hat{B}^\dagger_{\mathbf{m},k_y}] \right) \sin(k_z z_\mathbf{m}). \tag{S11}$$

In this notation, $\mathbf{m} = \{n_x, n_z\}$ specifies the lattice site by its in-plane index n_x and layer index n_z. The spatial location of the $\mathbf{n}$ site in the quantization direction $\vec{z}$ is written as $z_\mathbf{n} \equiv z_\mathbf{m} = (n_x - \frac{N_x}{2})b_x + (n_z a_z + \frac{L_z}{2})$. Here a_z is the inter-layer spacing and L_z is the distance between the two mirrors. This transformation allow us to write the full Hamiltonian into two non-interacting parts as

$$\hat{H}_{\text{LM}} = \hat{\mathcal{H}}_{\text{AX}}(\{\hat{X}_{\mathbf{k}_\parallel,n_z}, \hat{A}_{\mathbf{k}_\parallel}\}) + \hat{\mathcal{H}}_{\text{BY}}(\{\hat{Y}_{\mathbf{k}_\parallel,n_z}, \hat{B}_{\mathbf{k}_\parallel}\}), \tag{S12}$$

where $\hat{\mathcal{H}}_{\text{AX}}(\{\hat{X}_{\mathbf{k}_\parallel,n_z}, \hat{A}_{\mathbf{k}_\parallel}\})$ and $\hat{\mathcal{H}}_{\text{BY}}(\{\hat{Y}_{\mathbf{k}_\parallel,n_z}, \hat{B}_{\mathbf{k}_\parallel}\})$ have identical structure. Therefore, the dynamics of the light-matter Hamiltonian is captured by only focusing on $\hat{\mathcal{H}}_{\text{AX}}(\{\hat{X}_{\mathbf{k}_\parallel,n_z}, \hat{A}_{\mathbf{k}_\parallel}\})$ which is expressed as

$$\hat{\mathcal{H}}_{\text{AX}} = \sum_{k_y} \hat{\mathcal{H}}^{k_y}_{\text{AX}} = \sum_{k_y} \left[\sum_{k_x} \left(\hat{A}^\dagger_{\mathbf{k}_\parallel} \hat{A}_{\mathbf{k}_\parallel} \omega_\mathbf{k} + \sum_{n_z} \hat{X}^\dagger_{\mathbf{k}_\parallel,n_z} \hat{X}_{\mathbf{k}_\parallel,n_z} \epsilon_{\mathbf{k}_\parallel} \right) + \Omega_0 \sum_{\mathbf{m}} \sin(k_z z_\mathbf{m}) \left(\hat{X}^\dagger_{\mathbf{m},k_y} \hat{A}_{n_x,k_y} + \hat{X}_{\mathbf{m},k_y} \hat{A}^\dagger_{n_x,k_y} \right) \right]. \tag{S13}$$

The corresponding light-matter coupling constant is defined as $\Omega_0 = \frac{\sqrt{\omega_0}\mu g}{\sqrt{N_z}}$ (we set $\sqrt{\omega_0}\mu g = 0.35$ eV). This formulation enables us to perform independent 2D simulations (spanning $\vec{x}$ and $\vec{z}$ directions) at each k_y, which can be combined to reconstruct the full 3D dynamics of the system.

S3 Light-Matter Hamiltonian in the Reciprocal Space and in Effective Bright-Layer Representation

To gain intuition into the polaritonic band structure modification under material tilt, we write the light-matter Hamiltonian in the reciprocal space. Specifically, we use $\hat{X}^\dagger_{\mathbf{m},k_y} = \frac{1}{\sqrt{N_x}} \sum_{k_x} e^{ik_x n_x a_x} \hat{X}_{\mathbf{k}_\parallel,n_z}$ and $\sin(k_z z_\mathbf{m}) = \frac{e^{ik_z z_\mathbf{m}} - e^{-ik_z z_\mathbf{m}}}{2i}$, and Fourier transform $\hat{A}_{\mathbf{m},k_y}$

and obtain

$$\hat{H}_{\text{int},\mathbf{k}} = \frac{\Omega_0}{2i} \sum_{n_z,\mathbf{k}_\parallel} \hat{X}^\dagger_{\mathbf{k}_\parallel,n_z} \left(\hat{A}_{\mathbf{k}_\parallel+\mathbf{\Delta k_x}} e^{i\phi_z} + \hat{A}_{\mathbf{k}_\parallel-\mathbf{\Delta k_x}} e^{-i\phi_z}\right) - \hat{Y}^\dagger_{\mathbf{k}_\parallel,n_z} \left(\hat{A}_{\mathbf{k}_\parallel+\mathbf{\Delta k_x}} e^{i\phi_z} + \hat{A}_{\mathbf{k}_\parallel-\mathbf{\Delta k_x}} e^{-i\phi_z}\right) + \text{h.c.} \tag{S14}$$

Here, $\mathbf{\Delta k_x} = \Delta k_x \vec{x} = k_z \sin(\theta)\vec{x}$ and $\phi_z = k_z\left(\frac{-N_x b_x + L_z}{2} + n_z a_z\right)$, with $b_x = a_x \sin(\theta)$. This illustrates that two photon modes differing by $\pm 2\mathbf{\Delta k_x}$ in the x component of their wavevectors (given a tilt along x) *effectively* couple to each other through their interaction with the exciton.

To provide an analytical understanding of how the polaritonic dispersion is modified in a multilayered setup, we consider the light-matter Hamiltonian in the bright layer formalism presented in our recent works [2, 3]. In this formalism, a multilayered material is described using an effective bright layer which encapsulates the dynamics. Starting from Eq. S13, we define, $\hat{X}_{\mathbf{m},k_y} = \frac{1}{\sqrt{N_x}} \sum_{kx} \hat{X}_{\mathbf{k}_\parallel} e^{ik_x n_x a_x}$, which expresses the excitonic operators in real space for a given k_y. We then introduce the bright-dark operators, $\hat{X}^\dagger_{n_x,k_y,B}$ as,

$$\hat{X}^\dagger_{n_x,k_y,B} = \frac{1}{\sqrt{\mathcal{N}_{n_x}}} \sum_{n_z} \hat{X}^\dagger_{\mathbf{m},k_y} \sin(k_z z_{\mathbf{m}}); \quad \hat{X}^\dagger_{n_x,k_y,D_\xi} = \frac{1}{\sqrt{\mathcal{N}_{n_x}}} \sum_{n_z} \hat{X}^\dagger_{\mathbf{m},k_y} \mathcal{C}_{\xi,n_z} \text{ where } D_\xi \in \{1,2,3,...N_z-1\} \tag{S15}$$

where the normalization constant ($\mathcal{N}_{n_x}$) is defined as $\mathcal{N}_{n_x} = \sum_{n_z} \sin^2(k_z Z_{\mathbf{m}})$ while the coefficients $\{\mathcal{C}_{\xi,n_z}\}$ satisfy $\sum_{n_z} \mathcal{C}_{\xi,n_z} \sin(k_z z_{\mathbf{m}}) = 0$ and $\sum_{n_z} \mathcal{C}^2_{\xi,n_z} = 1$. Using these bright-dark excitonic operators, we write

$$\hat{\mathcal{H}}_{\text{AX}} = \sum_{\mathbf{k}_\parallel} \hat{A}^\dagger_{\mathbf{k}_\parallel} \hat{A}_{\mathbf{k}_\parallel} \omega_{\mathbf{k}} + \sum_{\mathbf{k}_\parallel} \left(\hat{X}^\dagger_{\mathbf{k}_\parallel,\text{B}} \hat{X}_{\mathbf{k}_\parallel,\text{B}} + \sum_{D_\xi} \hat{X}^\dagger_{\mathbf{k}_\parallel,D_\xi} \hat{X}_{\mathbf{k}_\parallel,D_\xi}\right)\epsilon_{\mathbf{k}_\parallel} + \Omega_0 \sum_{n_x,k_y} \sqrt{\mathcal{N}_{n_x}} \left(\hat{X}^\dagger_{n_x,k_y,\text{B}} \hat{A}_{n_x,k_y} + \hat{X}_{n_x,k_y,\text{B}} \hat{A}^\dagger_{n_x,k_y}\right). \tag{S16}$$

Since the dark excitonic modes are decoupled from the bright excitonic modes as well as any photonic degrees of freedom, we drop them to obtain the effective bright layer light-matter Hamiltonian $\hat{\mathcal{H}}^B_{\text{AX}}$ written as

$$\hat{\mathcal{H}}_{\text{AX}} \mapsto \hat{\mathcal{H}}^B_{\text{AX}} = \sum_{\mathbf{k}_\parallel} \hat{A}^\dagger_{\mathbf{k}_\parallel} \hat{A}_{\mathbf{k}_\parallel} \omega_{\mathbf{k}} + \sum_{\mathbf{k}_\parallel} \left(\hat{X}^\dagger_{\mathbf{k}_\parallel,\text{B}} \hat{X}_{\mathbf{k}_\parallel,\text{B}}\right)\epsilon_{\mathbf{k}_\parallel} + \Omega_0 \sum_{n_x,k_y} \sqrt{\mathcal{N}_{n_x}} \left(\hat{X}^\dagger_{n_x,k_y,\text{B}} \hat{A}_{n_x,k_y} + \hat{X}_{n_x,k_y,\text{B}} \hat{A}^\dagger_{n_x,k_y}\right). \tag{S17}$$

S4 Quantum Dynamical Approach

In this work the following light-matter Hamiltonian is simulated (assuming a simple dispersion-less excitonic sub-system):

$$\hat{\mathcal{H}}^{k_y}_{\text{AX}} = \sum_{\mathbf{k}_\parallel} \hat{A}^\dagger_{\mathbf{k}_\parallel} \hat{A}_{\mathbf{k}_\parallel} \omega_{\mathbf{k}} + \sum_{\mathbf{m}} \hat{X}^\dagger_{\mathbf{m},k_y} \hat{X}_{\mathbf{m},k_y} \epsilon_0 + \Omega_0 \sum_{\mathbf{m}} \sin(k_z z_{\mathbf{m}}) \left(\hat{X}^\dagger_{\mathbf{m},k_y} \hat{A}_{n_x,k_y} + \hat{X}_{\mathbf{m},k_y} \hat{A}^\dagger_{n_x,k_y}\right) \equiv \hat{H}_p + \hat{H}_e + \hat{H}_{e-p} \tag{S18}$$

This Hamiltonian is evolved using the time-dependent Schrödinger equation written as $i|\dot{\Psi}(t)\rangle = \hat{H}^{k_y}_{AX}|\Psi(t)\rangle$. In this work, we confine the exciton-polariton dynamics to the single excited subspace, such that

$$|\Psi(t)\rangle = \left(\sum_{k_x} c_{k_x}(t) \hat{A}^\dagger_{k_x} + \sum_{n_x,n_z} c_{n_x,n_z}(t) \hat{X}^\dagger_{n_x,n_z}\right)|\bar{0}\rangle = \sum_k c_{k_x}(t)|1_k\rangle + \sum_{n_x,n_z} c_{n_x,n_z}(t)|n_x,n_z\rangle \tag{S19}$$

where $c_{k_x}(t)$ and $c_{n_x,n_z}(t)$ are time-dependent coefficients. For simplicity, we have introduced the compact representation $|1_k\rangle \equiv \hat{A}^\dagger_{k_x}|\bar{0}\rangle$ and $|n_x,n_z\rangle \equiv \hat{X}^\dagger_{n_x,n_z}|\bar{0}\rangle$, with $|\bar{0}\rangle$ as the ground (or vacuum) state of the system representing no photons and no excitations in the material. Simulating the quantum dynamics via direct diagonalization of this system is computationally expensive, with $N_z \gg 1$ being intractable. To resolve this issue we use a split-operator approach where a single-step evolution (δt) of $|\psi(t)\rangle$ is written as

$$|\Psi(t+\delta t)\rangle = \hat{U}_{\text{cFT}} \cdot e^{-i[\hat{U}^\dagger_{\text{B}} \cdot \hat{H}_{e-p} \hat{U}_{\text{B}}]\delta t} \cdot \hat{U}^\dagger_{\text{cFT}} \cdot e^{-i(\hat{H}_e + \hat{H}_p)\delta t}|\Psi(t)\rangle. \tag{S20}$$

where $U^\dagger_{\text{cFT}} = \sum_{k_x,n_x} \frac{1}{\sqrt{N_x}} e^{ik_x n_x a_x}|1_{n_x}\rangle\langle 1_{k_x}|$ is a Fourier transformation operator that operates on the the photonic part of the wave function. Importantly, $\hat{U}^\dagger_{\text{B}}$ is a is the transformation to the bright/dark state representation that block diagonalizes the light-matter coupling part [2] ($\hat{H}_{e-p}$), which is defined as

$$\hat{U}^\dagger_B = \sum_{n_x} \left[\frac{1}{\sqrt{\mathcal{N}_{n_x}}} \sum_{n_z} \sin(k_z z_m)|n_x,B\rangle\langle n_x,n_z| + \sum_{\ell=1}^{N_z-1} \sum_{n_z} Q_{n_x,n_z,\ell}|n_x,D_\ell\rangle\langle n_x,n_z|\right]. \tag{S21}$$

Mixed Quantum-Classical Dynamics. To include the exciton-phonon coupling effect in the exciton-polariton dynamics, we utilize a mixed quantum-classical approach, namely mean-field ehrenfest [4, 5, 6, 7] method. In this scheme, the excitonic-photonic degrees of freedom are treated quantum mechanically using the following 1D generalized Holstein-Tavis-Cummings Hamiltonian given by

$$\begin{aligned}\hat{H}_{\mathrm{HTC}} &= \hat{\mathcal{H}}_{\mathrm{AX}}^{k_y=0} + \sum_{\mathbf{m}} \hat{b}_{\mathbf{m}}^{\dagger}\hat{b}_{\mathbf{m}}\omega_{\mathrm{b}} + \frac{\gamma}{\sqrt{2\omega_{\mathrm{b}}}}\sum_{\mathbf{m}} \hat{X}_{\mathbf{m},0}^{\dagger}\hat{X}_{\mathbf{m},0}(\hat{b}_{\mathbf{m}}^{\dagger}+\hat{b}_{\mathbf{m}}), \\ &= \hat{\mathcal{H}}_{\mathrm{AX}}^{k_y=0} + \frac{1}{2}\sum_{\mathbf{m}}\left(\hat{p}_{\mathbf{m}}^2 + \omega_b^2\hat{q}_{\mathbf{m}}^2\right) + \gamma\sum_{\mathbf{m}}\hat{q}_{\mathbf{m}}\hat{X}_{\mathbf{m},0}^{\dagger}\hat{X}_{\mathbf{m},0}\,. \end{aligned} \tag{S22}$$

The nuclear (phonon) coordinates are evolved (quasi) classically, following the equations of motion

$$\dot{p}_{\mathbf{m}}(t) = -\left\langle \Psi(t)\middle|\frac{\mathrm{d}\hat{H}_{\mathrm{HTC}}}{\mathrm{d}q_{\mathbf{m}}}\middle|\Psi(t)\right\rangle, \quad \dot{q}_{\mathbf{m}}(t) = p_{\mathbf{m}}(t)\,. \tag{S23}$$

S5 Evaluating Time-Independent Phase Difference

The light–matter moiré effect (LMME) can be used for coherent frequency conversion. We initialize the system in a photonic state $\hat{A}_{\mathbf{k}_{\|}}^{\dagger}|\bar{0}\rangle$ and $\hat{A}_{\mathbf{k}'_{\|}}^{\dagger}|\bar{0}\rangle$, and obtain an output at $\hat{A}_{\mathbf{k}_{\|}+2\Delta k}^{\dagger}|\bar{0}\rangle$ and $\hat{A}_{\mathbf{k}'_{\|}+2\Delta\mathbf{k}}|\bar{0}\rangle$. For coherent frequency conversion, we prepare an initial superposition of two single-photon modes with a relative phase (ϕ), given by

$$|\Psi(0)\rangle = \frac{1}{\sqrt{2}}\left[\hat{A}_{\mathbf{k}_{\|}}^{\dagger} + e^{i\phi}\hat{A}_{\mathbf{k}'_{\|}}^{\dagger}\right]|\bar{0}\rangle. \tag{S24}$$

The time-dependent input and output phase shifts are calculated using

$$\phi_{\mathrm{input}}(t) = \arg\left[\frac{\langle\bar{0}|\hat{A}_{\mathbf{k}_{\|}}|\Psi(t)\rangle}{\langle\bar{0}|\hat{A}_{\mathbf{k}'_{\|}}|\Psi(t)\rangle}\right],\ \phi_{\mathrm{output}}(t) = \arg\left[\frac{\langle\bar{0}|\hat{A}_{\mathbf{k}_{\|}+2\Delta\mathbf{k}}|\Psi(t)\rangle}{\langle\bar{0}|\hat{A}_{\mathbf{k}'_{\|}+2\Delta\mathbf{k}}|\Psi(t)\rangle}\right]. \tag{S25}$$

To extract the static (time-independent) phase shifts, $\phi_{\mathrm{input}}(t\to 0)$ and $\phi_{\mathrm{output}}(t\to 0)$, we determine the y-intercept of the linear fit to $\phi_{\mathrm{input/output}}(t)$ in the small interval $\Delta t = t_2 - t_1$ (see Fig. S1c). Explicitly,

$$\phi_{\mathrm{input/output}}(t\to 0) = \phi_{\mathrm{input/output}}(t_1) - t_1\times\Delta\phi_{\mathrm{input/output}}, \tag{S26}$$

$$\Delta\phi_{\mathrm{input/output}} = \frac{\phi_{\mathrm{input/output}}(t_1) - \phi_{\mathrm{input/output}}(t_2)}{\Delta t}, \tag{S27}$$

In this work, we used $t_1 = 17$ a.u. and $t_2 = 22$ a.u.

References

[1] Sun, K., Du, M. & Yuen-Zhou, J. Exploring the delocalization of dark states in a multimode optical cavity. *The Journal of Physical Chemistry C* **129**, 9837–9843 (2025).

[2] Koshkaki, S. R., Manjalingal, A., Blackham, L. & Mandal, A. Exciton-polariton dynamics in multilayered materials. *arXiv preprint arXiv:2502.12933* (2025).

[3] Mandal, A. *et al.* Microscopic theory of multimode polariton dispersion in multilayered materials. *Nano Letters* **23**, 4082–4089 (2023).

[4] Hoffmann, N. M., Schäfer, C., Rubio, A., Kelly, A. & Appel, H. Capturing vacuum fluctuations and photon correlations in cavity quantum electrodynamics with multitrajectory ehrenfest dynamics. *Physical Review A* **99**, 063819 (2019).

[5] Ghosh, P., Manjalingal, A., Wickramasinghe, S., Koshkaki, S. R. & Mandal, A. Mean-field mixed quantum-classical approach for many-body quantum dynamics of exciton-polaritons. *arXiv preprint arXiv:2505.04044* (2025).

[6] Li, T. E., Chen, H.-T. & Subotnik, J. E. Comparison of different classical, semiclassical, and quantum treatments of light–matter interactions: Understanding energy conservation. *Journal of Chemical Theory and Computation* **15**, 1957–1973 (2019).

[7] Crespo-Otero, R. & Barbatti, M. Recent advances and perspectives on nonadiabatic mixed quantum–classical dynamics. *Chemical reviews* **118**, 7026–7068 (2018).
